# Incidence of postoperative week 1 management changes after resident-performed phacoemulsification cataract surgery

**DOI:** 10.1186/s12886-021-02238-1

**Published:** 2022-01-08

**Authors:** Michael Bouaziz, Max Schlesinger, Joann J. Kang, Gene Kim

**Affiliations:** Department of Ophthalmology and Visual Sciences, Montefiore Medical Center, Albert Einstein College of Medicine, Bronx, NY USA

**Keywords:** Cataract extraction, Phacoemulsification, Resident-performed surgery, Post-operative outcomes

## Abstract

**Background:**

The goal of this study was to investigate the incidence of departures from routine care at the postoperative week 1 (POW1) visit following uneventful resident-performed cataract surgery in asymptomatic patients who had a normal postoperative day 1 (POD1) examination.

**Methods:**

A retrospective chart review of phacoemulsification surgeries performed by the senior resident class at Montefiore Medical Center between June 20, 2018 and April 1, 2019 was performed. The most recent preoperative visit note, operative report**,** POD1 visit note, and POW1 visit note were evaluated and variables were recorded. Exclusion criteria consisted of any complications that would have necessitated close follow-up and a POW1 visit, whether discovered preoperatively, intraoperatively, at the POD1 visit, or leading up to the POW1 visit. The primary outcome measure was the incidence of unanticipated management changes at the POW1 visit following resident-performed cataract surgery.

**Results:**

The charts of 292 surgical cases of 234 patients that underwent phacoemulsification with intraocular lens implantation were reviewed. 226 cases (77%) had an uncomplicated pseudophakic fellow-eye history, with a routine surgery, and POD1 examination. 19 of these patients had symptomatic presentations at the POW1 timepoint, and an additional 30 had no POW1 visit at all. In total, 177 cases were included in the study, and only 4 of these cases (2.3%) had an unexpected management change at the POW1 visit.

**Conclusions:**

Asymptomatic patients who underwent uncomplicated cataract surgeries performed by resident surgeons followed by a routine POD1 visit had a low incidence of unexpected management changes at the POW1 visit. These results suggest that regularly scheduled POW1 visits could potentially be omitted for patients deemed to be at low risk for complications, and instead performed on an as-needed basis.

## Background

In addition to providing care for large patient populations, academic teaching hospitals have the added obligation of training the future generation of physicians. Notwithstanding this additional responsibility, teaching hospitals, which can be defined by the presence of residents in training, have been shown to fare better in overall quality of care than nonteaching hospitals [1, 2]. Previous studies have found only small increases in complication and morbidity rates for different surgical procedures, with the overall consensus being that resident procedural involvement was safe [3, 4]. Specifically regarding resident involvement in cataract surgery, one group found no significant difference between resident and attending cataract surgeon outcomes in relation to postoperative complication rates [5]. Nonetheless, the data of resident outcomes in specific areas of comprehensive cataract treatment are lacking.

Cataract extraction with intraocular lens (IOL) implantation is the most frequently performed operation covered under the Medicare Part B insurance plan [6]. Given that academic teaching centers perform a substantial amount of cataract extractions with senior residents serving as the primary surgeons, the elucidation of this data is a critical step towards understanding the value of care provided. This is especially important considering that the overall disbursement of cataract procedures was reported to be the largest expenditure for any surgery covered under Medicare Part B, totaling $2.1 billion in 2009 and accounting for 1.8% of total costs covering Part B beneficiaries [7].

Following the performance of cataract surgery, physicians typically schedule their patients for three routinely timed follow-up visits: one at postoperative day 1 (POD1), another at postoperative week 1 (POW1), and a final visit 1 month after the surgery. While the first and third visits are deemed to be essential check-points for the monitoring of adequate progression following the procedure, the utility of the visit at POW1 has frequently been brought into question. Refraining from explicitly articulating the informally agreed upon 3-visit regimen, the Cataract in the Adult Eye Preferred Practice Pattern set forth the recommendation that patients have their first visit within 48 h in order to ensure appropriate recovery from surgery and an additional visit between 1 and 4 weeks after surgery for a more stabilized manifest refraction measurement [8].

Although there is limited evidence for the ideal postoperative visit schedule, we are able to quantifiably analyze the value of the POW1 visit by examining the rate at which departures from routine postoperative care occur. A POW1 visit is indisputably required if there was a complicated cataract extraction or a clinically significant presentation that already required careful monitoring by POD1. However, whether it is cost-effective for an asymptomatic patient with an uncomplicated ocular history, uncomplicated surgery, and an uneventful POD1 visit to come back for a week 1 check-in, is important to understand on the resident level at academic teaching hospitals. In doing so, we can achieve a more data-driven post-operative schedule that has the ability to reduce costs for both the healthcare system and for patients who are at a low risk for any potential unanticipated management change.

Departures from routine care, or unexpected management changes, can be defined as either a requirement for additional procedures, a need for referral to a specialty service, or a necessary change in postoperative drops. If a specific subset of patients has a minimal likelihood of requiring such an unexpected management change, then the removal of the week 1 visit as part of the routine protocol could have a substantial impact on healthcare costs. Instead of scheduled visits, these patients can be provided with adequate perioperative education along with an available line of communication to schedule an unplanned visit if any symptoms or complications do develop. Further, although the occurrence of complications is something that is inevitable, the timing of their pathologic manifestation is highly variable. It is therefore difficult to construct a schedule with visits whose purpose it is to detect these potential complications in a pre-symptomatic cohort of patients. A previous study has already shown that the POW1 visit in attending-performed cataract extractions has little function in a specific cohort of patients, shedding light on the potential for a more standardized and value-driven follow-up schedule [9]. The purpose of our study was to examine their findings within the context of resident-performed cataract surgeries; before established protocols are modified on an official level, we found it vital to establish whether it is safe to do so with ophthalmologists-in-training.

## Methods

This is a retrospective chart review of 234 patients who underwent cataract extraction by phacoemulsification with intraocular lens implantation at the resident cataract surgery service of Montefiore Medical Center between June 20, 2018 and April 1, 2019. The study was done in accordance with the Health Insurance Portability and Accountability Act. All experimental protocols were approved by the Institutional Review Board of Montefiore Medical Center and the Albert Einstein College of Medicine, and a waiver of informed consent was granted given the retrospective nature of our study.

The electronic medical records of Montefiore Medical Center were searched to identify patients who underwent cataract extraction according to the Classification of Procedural Terminology codes 66,982 (extracapsular cataract extraction with insertion of intraocular lens prosthesis, complex) and 66,984 (extracapsular cataract extraction with insertion of intraocular lens prosthesis). A chart review evaluating the most recent preoperative visit note, the operative report, and visit notes from POD1 and POW1 was then conducted and variables were recorded. The timeframe of the POW1 visit was defined as any visit that occurred between post-operative days 5 and 14.

Exclusion criteria consisted of findings that were present at each of these four different timepoints: preoperatively, intraoperatively, at the POD1 visit, or at the POW1 visit. Patients were excluded at the preoperative timepoint if they had a previous history of steroid intraocular pressure (IOP) response, persistent corneal edema, or rebound iritis following cataract surgery in the fellow eye. Intraoperative complications that led to exclusion included posterior capsular ruptures, anterior capsular rents (defined as any violation of the anterior capsule resulting in a discontinuous capsulorrhexis), performance of an anterior vitrectomy, zonular dehiscence, placement of a capsular tension ring, placement of the intraocular lens in the anterior chamber or sulcus, dropped nuclear fragments, or the performance of a concurrent vitreoretinal procedure. Patients were excluded at the POD1 timepoint if any unexpected clinical findings were noted that required a change in the standard post-operative care regimen of the primary surgeon. These included elevated IOP in the operative eye (defined as IOP ≥21 mmHg in patients with a history of glaucoma, ocular hypertension, or glaucoma suspect; or IOP ≥30 mmHg in patients without such history), intraocular lens out of position, severe corneal edema, corneal epithelial defects, a wound leak, a retained lens fragment, the performance of an anterior chamber paracentesis, or any change in the type or frequency of post-operative drops compared with the standard regimen. Patients were excluded at the POW1 timepoint if they presented with subjective ocular pathology-related symptoms either before or at the visit, including eye pain, redness, flashes, floaters, or decreased vision. Any patients that did not have a documented POD1 visit or a visit within the POW1 timeframe were also excluded.

The primary outcome measure of this study was the incidence of departures from routine care, or unexpected management changes, at the POW1 visit. Specifically, unexpected management changes consisted of a requirement for additional procedures other than suture removal, a need for referral to a specialty service, or an unplanned change in the frequency or type of postoperative drops.

## Results

Overall, the charts of 292 surgical cases of 234 patients that underwent phacoemulsification with intraocular lens implantation were reviewed. A total of 115 eyes were excluded; 4 eyes due to concerns from the contralateral eye at the preoperative timepoint, 18 due to intraoperative complications, 44 due to concerns at the POD1 timepoint, and 49 due to concerns at the POW1 timepoint (Table 1).

**Table 1:** Exclusion criteria for patients at each timepoint

Timepoint	Findings	Number of Patients Excluded
Preoperative (previous surgery in contralateral eye)	Elevated IOP	2
	Persistent corneal edema	1
	Rebound iritis	1
Intraoperative	Anterior chamber/sulcus IOL	12
	Posterior capsular rupture	11
	Anterior vitrectomy	11
	Zonular weakness	6
	Anterior capsular rent	2
POD1	Elevated IOP	24
	Severe corneal edema	7
	Corneal epithelial defect	3
	Wound leak	2
	Change in frequency of drops	2
	IOL malposition	1
	No visit	8
POW1	Symptoms	19
	No visit	30

Overall, 177 eyes of 145 patients were included in the study, with a mean age of 70.4 and 63% of the study population being female (Table 2). In relation to the preoperative characteristics, 41.8% of eyes had contralateral pseudophakia, 14.7% had a previous history of a glaucoma-related diagnosis 49.7% had diabetes, and 6.2% had a history of Flomax use (Table 3). Two patients had undergone previous intraocular surgeries, both of which had been pars plana vitrectomies.

**Table 2:** Demographic characteristics of eyes included in study

Total eyes included	177
Total patients included	145
Mean age (years)	70.4 ± 8.9
Age range (years)	45–95
Female (% eyes)	63.0
Race (% eyes)	
African American	26.6
Asian	13.0
Hispanic	55.4
White	2.2
Other	2.8

**Table 3:** Preoperative characteristics of eyes included in study

Characteristic	Number of eyes
Contralateral pseudophakia	74 (41.8%)
Diabetes	88 (49.7%)
Flomax use	11 (6.2%)
Prior intraocular surgery	2 (1.1%)
Glaucoma diagnosis (% eyes)	
Glaucoma suspect	15 (8.5%)
Pseudoexfoliation	5 (2.8%)
Primary angle closure glaucoma	4 (2.3%)
Ocular hypertension	2 (1.1%)
None	151 (85.3%)

Of the 177 eyes included in the study, 4 eyes (2.3%) had a departure from routine care following an asymptomatic presentation at the POW1 visit (Table 4). The most common unexpected management change was the addition of a hypertonic saline agent in 2 cases that had severe corneal edema. One patient underwent a wound revision performed in the operating room after the identification of a wound leak on exam. Another had the addition of an IOP-lowering agent due to an IOP of 30 mmHg with vitreous noted in the anterior chamber. This patient had no previous history of glaucoma, glaucoma suspect, or ocular hypertension, nor a history of a steroid IOP response after cataract surgery in the fellow eye. 25% of these patients had a history of diabetes or Flomax use. None of the 14.7% of patients with a prior history of glaucoma-related diagnoses had an unexpected management change at the POW1 visit.

**Table 4:** Postoperative week 1 unexpected management changes in asymptomatic patients following cataract surgery

Patient	Asymptomatic Presentation	Management Change
#1	Severe corneal edema	Addition of hypertonic drops
#2	Severe corneal edema	Addition of hypertonic drops
#3	Wound leak	OR revision
#4	Elevated IOP	Addition of IOP drops

Of note, the fellow eye was pseudophakic in 74 of the 177 eyes included in the study. Of the 103 phakic fellow eyes that were eligible for cataract surgery in the future, 9 eyes (8.7%) were scheduled for surgery at the POD1 visit and 30 eyes (29.1%) were scheduled at the POW1 visit (Table 5).

**Table 5:** Eligible eyes scheduled for cataract surgery during the postoperative course

Fellow eye phakic status	Number of eyes
Pseudophakic	74
Phakic	103
Surgery not scheduled	64 (62.1%)
Surgery scheduled at POD1	9 (8.7%)
Surgery scheduled at POW1	30 (29.1%)

## Discussion

The overall incidence of unexpected management changes in asymptomatic patients with an uneventful perioperative history, routine cataract surgery, and unremarkable POD1 visit was 2.3%. This finding is consistent with those found in previous studies [9–11]. However, our study was the first to specifically analyze data in resident-performed phacoemulsification with intraocular lens implantation. Previous findings have suggested that given the low incidence of management changes, providers can potentially exclude a specific cohort of patients from having this routine follow-up appointment. Nonetheless, standards of care are both practiced by attendings and taught to residents in academic teachings hospitals. If exclusion of the POW1 visit were to be implemented as a standardized practice pattern, we found it vital to identify whether it could also be a pragmatic and safe recommendation for resident-performed cataract surgeries. This is an especially important consideration given that the volume of resident-performed cataract surgeries continues to rise as academic teaching hospitals competitively provide their trainees with as many surgical cases as possible. We subsequently sought out to corroborate the evidence presented by previous studies with outcomes in resident-performed surgeries. Our findings are clinically significant, as any future recommended changes in the scheduled visit regimen in the postoperative period can safely be applied to both experienced attendings and residents in training.

In an older study that investigated cases of uncomplicated phacoemulsification, a clinical intervention rate of 2.8% was identified for all routine follow-up visits within a 120-day postoperative visit timeline [10]. This led the group to conclude that the most beneficial outcome of scheduled regimens is mutual reassurance. More recently, a group from Sweden conducted a prospective study in which they did not schedule postoperative visits for patients without any ocular comorbidities or surgical complications [11]. In doing so, they identified no significant difference in serious postoperative complications when compared to patients who received scheduled follow-up visits, which would indicate that it is safe for patients with uneventful cataract extractions to not have planned postoperative appointments. Their study, however, noted that the potential recommendation of no planned postoperative visits did not apply to ophthalmology residents. Although our study specifically studied the outcomes of management changes at the POW1 visit, we have shown here that the incidence of identifiable complications at this visit is comparably low in resident-led cases.

Also focusing specifically on the POW1 visit, another study that included cases from an experienced group of 10 cataract surgeons determined the overall incidence of unexpected management changes in asymptomatic patients with uneventful cases to be 0.9% [9]. The same team of investigators subsequently established a method of sensitively predicting patients who would not require any management changes at their POW1 visit depending on how they responded to a clinical set of ocular-pathology related questions [12]. Although this group’s data did not apply to resident-performed cases, our data substantiates their findings and can broaden the applicability of their standardized questionnaire to be used in resident-led cases as well.

Of note, a previous study done by a group in New Zealand whose data collection was performed more than 20 years ago determined the incidence of detected complications at the POW1 visit to be 4.1%, a value significantly higher than our reported findings [13]. Like our study, 38% of all of the cataract surgeries performed in their study were done by “trainee surgeons”, the equivalent of a resident in the United States. These conflicting findings can be attributed to a few different factors. In conducting their study, the group from New Zealand had no exclusion criteria and included patients that had complex ocular histories, intraoperative complications, POD1 complications, and patients that presented with ocular symptoms before or at the POW1 visit. For instance, 10.1% of the subjects included in their study had significant complications on POD1, with the most common clinical problem being elevated intraocular pressure [13]. While this supports the American Academy of Ophthalmology’s recommendation that all patients should be examined within 48 h of the operation, it confounds whether patients with no reported complications should be examined at the POW1 visit. After stratifying their data, they found that approximately 8% of patients with either a pre- or intraoperative risk factor had identifiable complications at the POW1 visit, confirming that individuals with ocular comorbidities require careful monitoring. Another important difference to consider is the time at which their study was conducted. Advancements in phacoemulsification since the time their analysis was performed may have naturally resulted in a decrease in post-operative complications requiring an unexpected management change.

Our study excluded patients with POD1 findings that would have necessitated close observation in the follow-up period. Overall, 39 of 292 cases (13.4%) were excluded from our study for this reason. The most common cause for exclusion in this cohort was an elevation in IOP (61.5%). This corroborates the guidelines proposed by the American Academy of Ophthalmology that patients should have their first visit following cataract extraction within 48 h of the procedure. However, no mention is made of a POW1 timepoint, and previous studies have exhibited the potential lack of utility of this visit for an asymptomatic subset of patients. Our study further adds strength to these findings by substantiating their outcomes with strictly resident-performed cataract surgeries in an academic teaching hospital. It also adds to previous studies that have shown no significant difference in intraoperative and postoperative complication rates between resident-performed and attending-performed cataract surgeries [5, 14].

Our study has a few notable limitations. Many attending physicians at our institution already exclude POW1 visits from their standard postoperative follow-up schedule, and we therefore did not directly compare the incidence of unexpected management changes in resident-performed cases to attending-performed ones. As mentioned previously, however, our findings are consistent with rates reported in other studies in the literature. Additionally, although our study was sufficiently powered to analyze the incidence of unexpected POW1 management changes, the sample size was not large enough to adequately study the risk factors that could potentially necessitate a POW1 visit. Further studies could be done to identify such risk factors.

## Conclusion

In conclusion, we found very low rates of unexpected management changes at POW1 visits following resident-performed cataract surgery in a specific subset of patients. Our findings are in agreement with other recent studies in the literature which suggest that this visit may no longer be an essential part of routine cataract follow-up. Having found that nearly 30% of eligible fellow-eye cataract surgeries were booked at the POW1 appointment, it appears that the greatest utility of this visit may lie in its potential for coordinating future care. Future studies aimed at establishing alternative means of post-operative follow-up in lieu of an in-person encounter, such as telemedicine visits or video calls, could be valuable in expanding on these findings.

## Data Availability

The datasets generated and analyzed during the current study are available from the corresponding author on reasonable request.

## Abbreviations

POW1: Postoperative week 1
POD1: Postoperative day 1
IOL: Intraocular lens
IOP: Intraocular pressure
